# The Chicken Chorioallantoic Membrane Tumor Assay as a Relevant In Vivo Model to Study the Impact of Hypoxia on Tumor Progression and Metastasis

**DOI:** 10.3390/cancers13051093

**Published:** 2021-03-04

**Authors:** Kelly Harper, Anna Yatsyna, Martine Charbonneau, Karine Brochu-Gaudreau, Alexis Perreault, Claudio Jeldres, Patrick P. McDonald, Claire M. Dubois

**Affiliations:** 1 Department of Immunology and Cell Biology, Université de Sherbrooke, Sherbrooke, QC J1H 5H3, Canada; kelly.harper@usherbrooke.ca (K.H.); Anna.Yatsyna@usherbrooke.ca (A.Y.); martine.charbonneau@usherbrooke.ca (M.C.); Karine.brochu-gaudreau@usherbrooke.ca (K.B.-G.); Alexis.perreault@usherbrooke.ca (A.P.); 2 Department of Surgery, Division of Urology, Université de Sherbrooke, Sherbrooke, QC J1H 5H3, Canada; claudio.jeldres@usherbrooke.ca; 3 Department of Medicine, Pulmonary Division, Université de Sherbrooke, Sherbrooke, QC J1H 5H3, Canada; patrick.mcdonald@usherbrooke.ca

**Keywords:** hypoxia, ChorioAllantoic Membrane, chick embryo, metastasis, tumor progression

## Abstract

**Simple Summary:**

Hypoxia is a negative prognostic factor known to be closely associated with tumor progression and metastasis. However, existing animal models with the ability to recreate the tumor hypoxic microenvironment have disadvantages that limit our ability to understand and target this pathological condition. The chicken ChorioAllantoic Membrane (CAM) assay is increasingly used as a rapid cost-effective drug-testing model that recapitulates many aspects of human cancers. Whether this model recreates the hypoxic environment of tumors remains understudied. Here, we demonstrate that the CAM model effectively supports the development of hypoxic zones in a variety of tumor types. Treatment of tumors with angiogenesis inhibitors or inducers significantly modulated the formation of hypoxic zones as well as tumor progression and metastasis. Our findings suggest that the CAM-based tumor model is a relevant in vivo platform to further understand the pathological responses to hypoxia and test therapeutic interventions aimed at targeting hypoxic cancers.

**Abstract:**

Hypoxia in the tumor microenvironment is a negative prognostic factor associated with tumor progression and metastasis, and therefore represents an attractive therapeutic target for anti-tumor therapy. To test the effectiveness of novel hypoxia-targeting drugs, appropriate preclinical models that recreate tumor hypoxia are essential. The chicken ChorioAllantoic Membrane (CAM) assay is increasingly used as a rapid cost-effective in vivo drug-testing platform that recapitulates many aspects of human cancers. However, it remains to be determined whether this model recreates the hypoxic microenvironment of solid tumors. To detect hypoxia in the CAM model, the hypoxic marker pimonidazole was injected into the vasculature of tumor-bearing CAM, and hypoxia-dependent gene expression was analyzed. We observed that the CAM model effectively supports the development of hypoxic zones in a variety of human tumor cell line-derived and patient’s tumor fragment-derived xenografts. The treatment of both patient and cell line-derived CAM xenografts with modulators of angiogenesis significantly altered the formation of hypoxic zones within the xenografts. Furthermore, the changes in hypoxia translated into modulated levels of chick liver metastasis as measured by Alu-based assay. These findings demonstrate that the CAM xenograft model is a valuable in vivo platform for studying hypoxia that could facilitate the identification and testing of drugs targeting this tumor microenvironment.

## 1. Introduction

The tumor microenvironment is increasingly recognized as a critical element for the development and progression of malignant tumors. This microenvironment is characterized by a network of stromal and immune cells, inadequate and heterogeneous vascularization, hypoxia, and acidic extracellular pH [1]. Among these, hypoxia has been widely associated with increased cell aggressiveness, metastasis, and resistance to treatment [2]. More specifically, tumor hypoxia drives genetic instability, selects for resistance to apoptosis, increases cell survival and angiogenesis, and alters tumor metabolism [3,4,5]. Hypoxia also affects tumor immune responses and reduces cell–cell attachment allowing cancer cells to migrate, thereby increasing the risk of both local invasion and metastatic spread [6,7,8]. Furthermore, the upregulation of multidrug resistance genes and increase in cellular acidification observed under hypoxic conditions contribute to the resistance to conventional cytotoxic therapies such as chemotherapy, radiotherapy, and immunotherapy [9,10,11]. Due to these profound effects on multiple aspects of carcinogenesis, hypoxia is considered one of the major factors for anti-cancer therapy failure and one of the strongest indicators of poor prognosis [12]. It is therefore not surprising to find that various strategies have been put forward to target hypoxic cells, including the development of hypoxia-activated prodrugs (HAPs), which are activated in low oxygen environments. Several HAPs have been developed but, despite encouraging results from preclinical studies, these drugs have performed disappointingly in clinical trials [13]. 

In vivo tumor models are essential for comprehending the dynamic process of human cancer progression, identifying therapeutic targets, and evaluating the efficacy of anti-tumor drugs. Therefore, preclinical models that allow the detection and assessment of tumor hypoxia play a critical role in both the validation and development of new hypoxia-targeting or -modifying therapies for their eventual adoption into routine clinical practice [12]. Currently, mouse xenograft models are the most widely used preclinical assays for testing hypoxia-targeting drugs. Tumor hypoxia has been observed in mouse models of osteosarcoma [14], fibrosarcoma [15], glioma [16], head and neck cancer [17], lung cancer [18], melanoma [19], prostate cell line-derived xenografts [20], and colorectal PDX tumors [21]. However, mouse xenograft models are costly, time-consuming assays that require large cohorts of animals. Several ethical issues are also to consider when using mice in research. For instance, some models of tumor hypoxia rely on invasive surgical intervention, such as vessel ligation or clamping of tumor blood supply, which can cause significant distress to the animal [22,23]. In addition, many preclinical studies using mouse PDX models failed to predict drug efficacy in patients [24]. Therefore, there is a need for alternative models for cancer research that reduce animal distress while increasing reproducibility and lowering costs [25].

The chicken ChorioAllantoic Membrane (CAM) model provides an attractive alternative in vivo model for cancer research. The CAM is a highly vascularized extra-embryonic membrane connected to the developing embryo through an easily accessible circulatory system. This model has many advantages compared to current in vivo models, notably its being cost-effective, rapid, and having fewer ethical issues, as the chick embryo is unable to perceive pain from the CAM tissue, which is not innervated [26]. Due to the immaturity of the chick embryo’s immune system, the CAM allows successful engraftment of a variety of foreign tissues such as tumor explants or cancer cell line suspensions [27]. The CAM assay has been widely used to study angiogenesis [28,29], and more recently, to assess invasion and metastasis in many cancer types including prostate, melanoma, ovarian, and head and neck cancers [30,31,32,33,34], and even evaluate the efficacy of anti-cancer drugs [35,36]. This model could therefore provide an interesting platform for preclinical characterization of novel anti-cancer drugs targeting hypoxia. For this purpose, however, the CAM xenograft model must recapitulate certain key aspects of the tumor microenvironment, including hypoxia. 

Here, we provide an original demonstration that the CAM model efficiently supports the development of hypoxic zones in a variety of human tumor cell line-derived xenografts as well as human clear cell Renal Cell Carcinoma (ccRCC) patient-derived xenografts. Treatment of xenografts with angiogenesis inhibitors or inducers significantly modulated the formation of hypoxic zones as well as tumor progression and metastasis. These findings suggest that the CAM xenograft model is a relevant platform to test therapeutic interventions targeting hypoxia that can be applied to various cancer types.

## 2. Materials and Methods

### 2.1. Reagents

VEGF was from Peprotech (Rocky Hill, NJ, USA, 450-32), Sorafinib was from Toronto research chemicals inc (North York, ON, Canada, S676850) Rhodamine-labeled LCA (lens culinaris agglutinin) was from Vector Laboratories (Burlingame, CA, USA, RL1042). Pimonidazole hydrochloride was purchased from Hydroxyprobe Inc. (Burlington, MA, USA, HP-100MG). Matrigel Basement Membrane Matrix was from Corning (Bedford, MA, USA, 356234).

### 2.2. Cell Culture

HT-1080 fibrosarcoma, PANC-1 pancreatic, HCT 116 colorectal, Caki-1 renal, and A549 lung cancer cell lines were obtained from the American Type Culture Collection (ATCC, Rockville, MD, USA). Cells were cultured in EMEM (HT-1080, A549) (320-005-CL), DMEM (PANC-1, HCT 116) (319-020-CL) or McCoys (Caki-1) (317-010-CL) medium (Wisent, St-Bruno, QC, Canada, 320-005-CL) supplemented with 10% fetal bovine serum (FBS, Gibco BRL, Burlington, ON, Canada, 12483-020) and 40 µg/mL of gentamicin (Wisent, St-Bruno, QC, Canada, 450-135-QL) in a humidified 95% air/5% CO_2_ incubator at 37 °C. All cell lines were routinely tested for mycoplasma using the MycoSEQ Mycoplasma Detection Kit (Thermo Fisher Scientific, Mississauga, ON, Canada).

### 2.3. ccRCC Patient Tumors

ccRCC patients’ tumor tissue was obtained from patients undergoing a nephrectomy at the Centre Intégré Universitaire de Santé et de Services Sociaux de l’Estrie (CIUSSS-Estrie). Patients were recruited in collaboration with Dr. Claudio Jeldres and Dr. Nadia Ekindi Ndongo. The project was approved by the Organizational Ethics Committee of the CIUSSS de l’Estrie-CHUS (Protocol # 2017-1524). All patients provided written informed consent. Fresh tumor tissues specimens from the kidneys of patients were collected in the pathology laboratory at the CIUSSS-Estrie. Samples were cut into 2 mm^3^ sections and immediately implanted on the CAM as described below.

### 2.4. Chorioallantoic Membrane Assays

Fertilized eggs from White Leghorn chicken were obtained from the Public Health Agency of Canada (Ottawa, ON, Canada). Protocols were approved by the Ethics Committee on Animal Research of the University de Sherbrooke (Protocol # 054-13), and all experimental procedures involving embryos were conducted in accordance with regulations of the Canadian Council on Animal Care. Chorioallantoic membrane (CAM) assays were performed as described [37,38], with the following modifications. At embryonic day (ED) 9, HT1080, PANC-1, HCT116, Caki-1, or A549 cell suspensions (3 × 10^5^ cells) were mixed (1:1) with standard Matrigel basement membrane matrix to prevent cell dispersion, in a total volume of 20 µL. The number of cells used for implantation was chosen based on preliminary concentration-dependent experiments where we found that high cell concentrations during the implantation procedure can result in a loss of cell viability. For patients’ tumor fragments, Matrigel and medium were mixed 1:1 in a total volume of 20 µL and added to the CAM prior to deposing the tumor fragment. When indicated, cell suspensions were mixed with VEGF prior to inoculation on the CAM and Sorafenib was added topically to the developing tumors every day. Assays to evaluate tumor growth and metastasis in chick embryos were performed as described [37,38]. Briefly, at ED16, chick embryos were sacrificed by decapitation. Tumors were removed and imaged and tumor volumes were calculated using the formula: (Dd^2^/2). Tumors were then either snap-frozen in liquid nitrogen, for future RNA extraction and PCR quantification, or frozen into OCT, for immunohistochemistry. Livers from the chick embryos were removed and immediately snap-frozen in liquid nitrogen and stored at −80 °C until DNA extraction. 

### 2.5. RNA/DNA Isolation and qPCR Analysis

Total RNA was extracted from frozen powdered tumors using Trizol reagent (Invitrogen, Waltham, MA, USA, 15596-018) and 1 μg of RNA was reverse transcribed to complementary DNA (cDNA) using QuantiTect reverse transcription kit (Qiagen, Mississauga, ON, Canada, 205314). The cDNA was then diluted in a 1:5 ratio in RNAse- and DNAse-free water. Genomic DNA was extracted from frozen powdered livers using DNAzol reagent according to the manufacturer’s instructions (Invitrogen, Waltham, MA, USA, 10503027).

Genomic DNA or cDNA was then analyzed by real-time PCR using a hot start SYBR Green qPCR Master Mix (BiMake, Houston, TX, USA). The following primer pairs were selected: *RPLP0* (sense 5′- GAT TAC ACC TTC CCA CTT GC -3′, and antisense 5′- CCA AAT CCC ATA TCC TCG TCC -3′), *CA9* (sense 5′- CCT CAA GAA CCC CAG AAT AAT GC -3′, and antisense 5′- CCT CCA TAG CGC CAA TGA CT -3′), *SLC2A1* (sense 5′- GGC CAA GAG TGT GCT AAA -3′, and antisense 5′- CTT CTT CTC CCG CAT CAT C -3′), *PDK1* (sense 5′- ACC AGG ACA GCC AAT ACA AG -3′, and antisense 5′- CCT CGG TCA CTC ATC TTC AC -3′), Chick *GAPDH* (sense 5′- GAG GAA AGG TCG CCT GGT GGA TCG -3′, and antisense 5′- GGT GAG GAC AAG CAG TGA GGA ACG -3′), and human *Alu* (sense 5′- ACG CCT GTA ATC CCA GCA CTT -3′, and antisense 5′- TCG CCC AGG CTG GAG TGC A-3′). Quantitative Real-Time PCR was performed on a Rotor-Gene 3000 (Corbett Research, Kirkland, QC, Canada). The cycling program was as follows: initial denaturation at 95 °C for 15 min, 35 amplification cycles with annealing T° of 55 °C for 30 s and final extension at 72 °C for 30 s. Relative changes in gene expression were calculated using the 2^−ΔΔCT^ method, and results are expressed as fold increase relative to the control condition set at 1 (vehicle-treated tumors). Human cells within chick embryo liver were detected by real-time Alu PCR as previously described [39], and relative changes in metastasis were then calculated as 2^−ΔΔCT^.

### 2.6. Immunohistochemistry

CAMs bearing tumors were intravenously injected with pimonidazole hydrochloride (1.5 mg/CAM) to detect hypoxia and Lens culinaris agglutinin (LCA) (0.05 mg/CAM) to label the CAM blood vessel network [40,41,42,43,44], 30 min before harvesting tumors. Tumors removed from the CAM were placed directly in the cryopreservative embedding media OCT compound (Electron Microscopy Sciences, Hatfield, PA, USA, 62550-01), immediately frozen in dry ice-cooled isopentane and kept at −80 °C until sectioning for immunostaining. Sections were fixed with PFA 2% for 10 min at 4 °C. Blocking and staining were performed in BSA 2% with 10% goat serum. Pimonidazole was detected with mouse antibody (Hypoxyprobe, 1:200) and goat anti-mouse Alexa Fluor 488 (Invitrogen). Nuclei were stained with 1 µg/mL DAPI (4,6-diamidino-2-phenylindole, Invitrogen, Molecular Probes, Eugene, OR, USA). Stained sections were visualized using an Axioskop 2 phase-contrast/epifluorescence microscope (Carl Zeiss Inc., Thornwood, NY, USA). 

### 2.7. Image Acquisition and Quantification of Hypoxia and Vascularization

For quantification of CAM tumors hypoxic fractions, five 7 µm sections spaced approximately 150 µm apart were analyzed per tumor. For quantification of vascularization, the LCA signal in 30 µm sections was analyzed. Images of whole tumor sections were captured using a Zeiss AxioImager M2 ApoTome microscope and analyzed using Imaris software. Hypoxic fraction results are expressed as the surface area occupied by pimonidazole staining (hypoxic area) in µm^2^, divided by the surface area occupied by DAPI staining (total surface area) in µm^2^. For quantification of vascularization, 10-point grid squares (100 μm × 100 μm) were analyzed for various sections of the tumor. The calculation of the number of anastomoses and capillaries per mm^3^ was performed as previously described [45]. To estimate the number of capillaries, the connectivity of the vascular node segment was estimated based on the number of branching points or nodes in the vascular network. 

### 2.8. Statistical Analysis

Sample sizes are denoted in figures or figure legends and refer to the number of tumors, unless otherwise noted. Statistical analysis was performed using Prism 8 Software (GraphPad, San Diego, CA, USA). Significance was determined using non-parametric *t* tests or one-way ANOVA, as indicated in figure legends. For all comparisons, a *p*-value of ≤0.05 was considered statistically significant. Data are expressed as mean +/− SEM. 

## 3. Results

### 3.1. The Chicken Chorioallantoic Membrane Assay Supports the Development of Hypoxic Zones in Cancer Cell Line-Derived Xenografts

**Figure 1:**
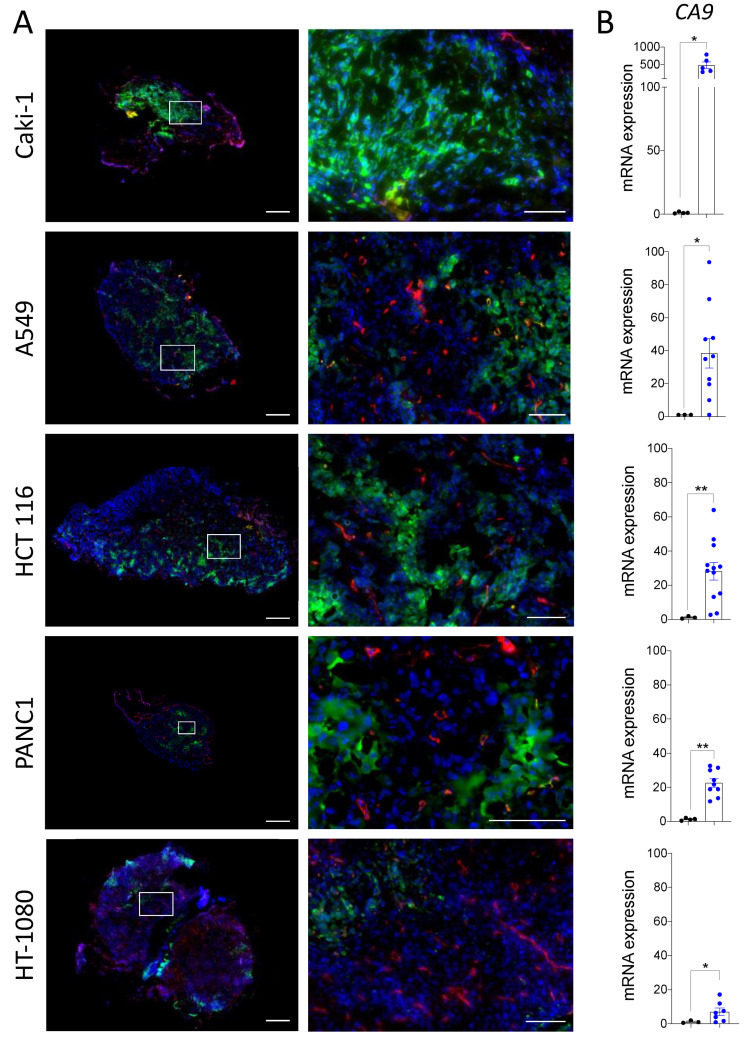
(**A**,**B**) HT-1080, HCT 116, A549, PANC-1, or Caki-1 cancer cell lines were inoculated onto ChorioAllantoic Membrane (CAM) and grown for 7 days. A mix of Lens culinaris agglutinin (LCA) (0.05 mg/CAM) and pimonidazole (1.5 mg/CAM) was injected into CAM vasculature prior to tumor xenograft extraction from the CAM. (**A**) Representative immunofluorescence images and corresponding zooms showing regions of hypoxia (pimonidazole; green) and vascularization (LCA; red) in tumor xenografts. Nuclei were stained with DAPI (blue). Scale bar = 500 µm. Zoomed area is outlined in white, scale bar = 100 µm. (**B**) qPCR analysis of mRNA isolated from HT-1080, HCT 116, A549, PANC-1, or Caki-1 cell lines in 2D culture or xenografts grown on CAM. Results are expressed as relative mRNA expression of *CA9* (CAIX) in tumor xenografts (blue dots) compared to corresponding cell lines in culture (black dots). RPLP0 was used to normalize the data. (cell lines in 2D culture *n* = 3, cell line-derived xenografts *n* = 6–13). Bars represent the mean ± SEM. The asterisks (*) correspond to *p* < 0.05 (*) and *p* < 0.01 (**), non-parametric *t* test.

To define whether the chicken chorioallantoic membrane (CAM) assay is a suitable in vivo model for studying the hypoxic microenvironment of solid tumors, we first characterized the presence of hypoxia in a variety of cancer cell line-derived xenografts grown on CAMs. Human cancer cell lines originating from diverse types of cancers, fibrosarcoma (HT-1080), pancreatic (PANC-1), colorectal (HCT 116), renal (Caki-1) and lung cancer (A549), were studied. All cell lines tested grew viable tumors irrigated by the CAM vasculature, as detected by the red LCA staining of blood vessels throughout the xenograft tissue sections (Figure 1A). We observed various hypoxic zones, identified by pimonidazole staining, in each of the cell line xenografts grown on the CAM. Caki-1-derived tumors displayed large zones of intense pimonidazole staining, A549, HCT 116, and PANC1 tumors all had numerous smaller zones of hypoxia, while HT-1080 tumors showed very few small zones of hypoxia. Notably, strong pimonidazole staining was observed in areas of the tumors with very few or no blood vessels, as visualized in the zoomed images (Figure 1A). QPCR analysis of carbonic anhydrase IX (CAIX) expression, a commonly used hypoxia marker [46,47], revealed elevated CAIX mRNA expression in the different cell line-derived CAM xenografts compared to the corresponding cells cultivated in vitro (Figure 1B). Furthermore, the relative pimonidazole staining intensity in cell line-derived xenografts appeared to be consistent with the level of CAIX mRNA expression with the following ranking: Caki-1 > A549 > HCT 116 > PANC1 > HT-1080. These results demonstrate that hypoxia is present, in varying degrees, within multiple cancer cell line-derived xenografts grown on the CAM.

### 3.2. Hypoxia in Tumors Grown on the CAM Can Be Modulated by Pro- and Anti-Angiogenic Treatments

**Figure 2:**
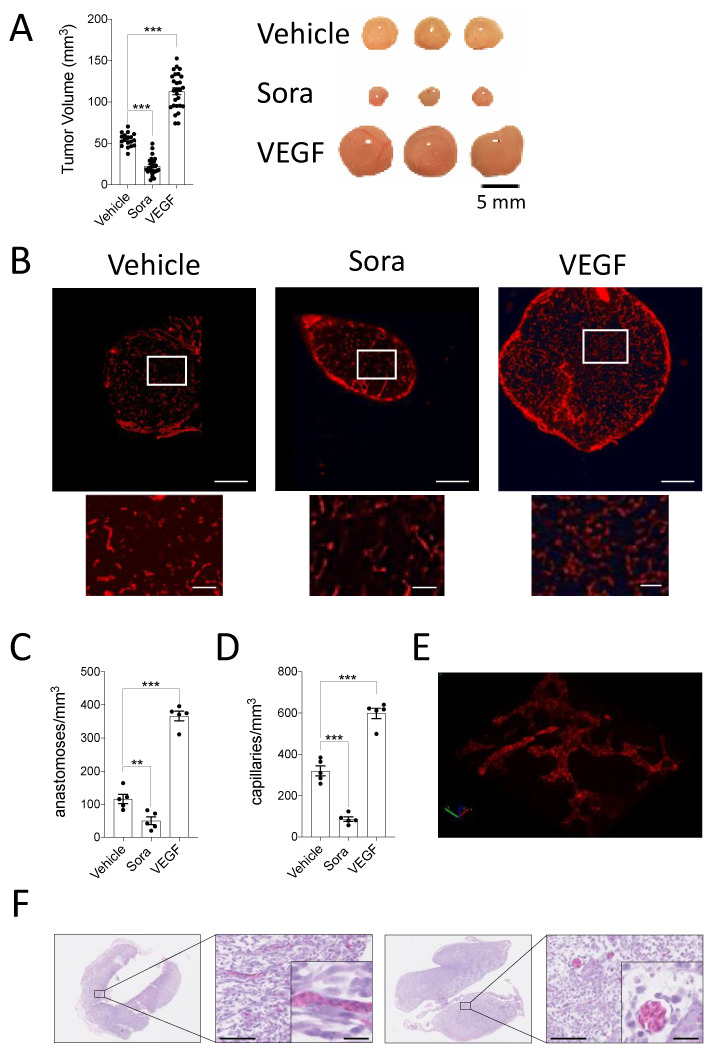
(**A**–**D**) HT-1080 cell suspensions were inoculated onto the CAM in the presence or absence of VEGF while Sorafenib (Sora) or DMSO (Vehicle) were added topically to the developing tumors every day. (**A**) Tumor volumes were calculated after 7 days of growth and representative images of tumors are shown (*n* = 18–27). (**B**–**D**) LCA was injected into CAM vasculature prior to tumor extraction. (**B**) Representative immunofluorescence images and corresponding zooms showing vascularization in the control and treated tumor xenografts (LCA; red). Scale bar = 500 μm. Zoomed area is outlined in white, scale bar =100 μm. (**C**,**D**) Quantification of the number of anastomoses (**C**), and capillaries (**D**) per mm^3^ are shown (*n* = 5). (**E**,**F**) 3D reconstruction of the CAM vasculature using Imaris Software (**E** (Video S1)) and representative H&E staining of HT1080 tumor xenografts cultivated on the CAM for 7 days (**F**). Higher magnification shows the presence of chicken nucleated erythrocytes. Scale bars, 100 μm. Inset scale bars, 25 μm. Bars represent the mean ± SEM. The asterisks (*) correspond to *p* < 0.01 (**), and *p* < 0.001 (***), one-way Anova.

Intratumoral hypoxia is a dynamic state that fluctuates according to the rapid cancer cell proliferation rate and the generation of new blood vessels within the tumor [48]. To evaluate whether the CAM xenograft model can reproduce this intratumoral hypoxic plasticity, we modulated the tumor xenograft vasculature using the anti- and pro-angiogenic treatments, sorafenib, and VEGF, respectively. First, the effect of these angiogenesis modulators on tumor growth and angiogenesis was examined. As expected, inhibiting angiogenesis with sorafenib reduced tumor volumes of HT-1080 CAM xenografts while the pro-angiogenic factor VEGF increased tumor volumes (Figure 2A). To confirm that the pro- and anti-angiogenic treatments were, in fact, modulating angiogenesis, blood vessels were visualized in frozen tissue sections of CAM HT-1080 xenografts treated with sorafenib or VEGF and injected into the CAM vasculature with the endothelium-labeling reagent, LCA. Immunofluorescent images of tumor xenografts revealed less abundant blood vessels in the sorafenib-treated tumors while a marked increase in the number of blood vessels was observed in the VEGF-treated condition (Figure 2B). 

To quantify these observations, the number of capillaries and the number of anastomoses, which are needed to form new flow channels for oxygen supply, were calculated [45,49]. Treatment with sorafenib significantly decreased the number of anastomoses per mm^3^ as well as the number of capillaries per mm^3^ in the xenografts. Conversely, a significant increase in these parameters was observed in VEGF-treated xenografts (Figure 2C,D). H&E images and high magnification zooms of the LCA-stained vasculature reveal the 3D vascular network, which is well-perfused with nucleated avian erythrocytes, suggesting their functionality (Figure 2E,F and Video S1).

**Figure 3:**
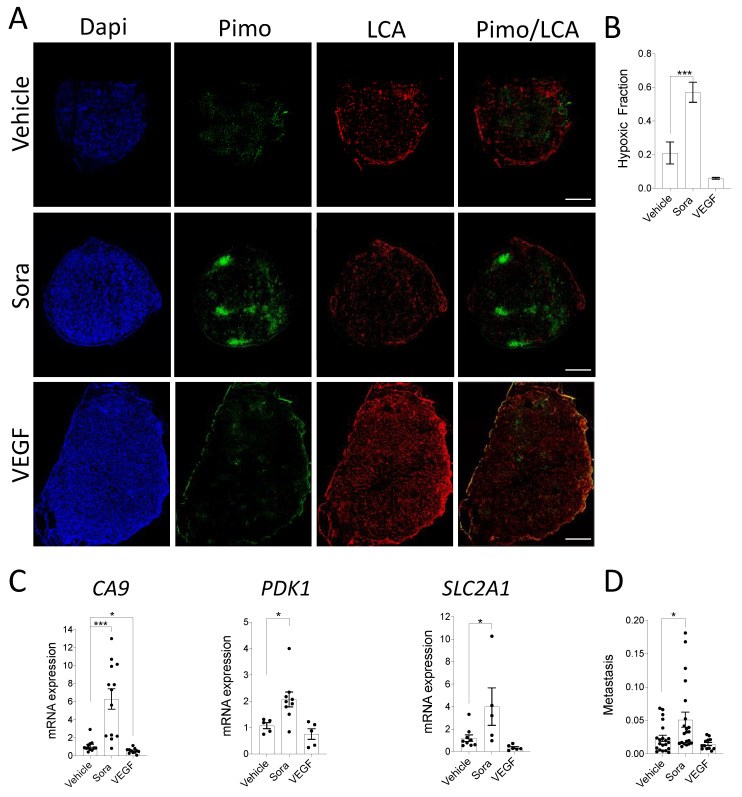
(**A**–**C**) HT-1080 cell suspensions were inoculated onto the CAM in the presence or absence of VEGF while Sorafenib (Sora) or DMSO (Vehicle) were added topically to the developing tumors every day. A mix of LCA and pimonidazole was injected into CAM vasculature prior to tumor extraction from the CAM. (**A**) Representative immunofluorescence images showing regions of hypoxia (pimonidazole; green) and vascularization (LCA; red) in control and treated tumor xenografts are shown. Nuclei were stained with DAPI (blue). Scale bar = 500 µm (**B**) The hypoxic fraction of control or treated tumor xenografts was quantified (*n* = 5–7). (**C**) Relative mRNA expression of *CA9*, *SLC2A1*(GLUT1), and *PDK1* in treated vs. control tumor xenografts is shown (*n* = 5–13). (**D**) Embryonic livers were harvested after 7 days of tumor growth for genomic DNA extraction. The ability of HT1080 cells to disseminate in embryonic livers was quantified as the relative amount of metastasis (Alu) normalized to the amount of host DNA (GAPDH). Metastasis per mm^3^ of original tumor is shown (*n* = 12–21). Bars represent the mean ± SEM. The asterisks (*) correspond to *p* < 0.05 (*) and *p* < 0.001 (***), one-way ANOVA.

To investigate the effect of these angiogenic modifiers on tumor hypoxia, CAMs were injected into the CAM vasculature with the hypoxia marker pimonidazole, and the blood vessel staining reagent LCA, and the resected tumors were submitted to histological examination. Untreated and VEGF-treated HT-1080 tumors displayed a weak and diffuse pimonidazole-stained pattern, whereas sorafenib-treated tumors showed multiple enhanced pimonidazole-stained regions (Figure 3A). The hypoxic areas were found in central and peripheral regions of the tumors, and co-localized with avascular or very low vascular density regions. Quantitative analysis of pimonidazole staining indicated that the hypoxic fraction was significantly higher in sorafenib-treated tumors, while a non-significant decrease was observed in tumors treated with VEGF (Figure 3B). Consistent with this observation, qPCR quantification of common hypoxia-induced genes showed that sorafenib treatment increased the expression of *CA9, SLC2A1* (GLUT1), and 3-phosphoisonitide-dependent protein kinase-1 (*PDK1*)*,* while VEGF decreased their expression (Figure 3C). We then evaluated the correlation between tumor size and the most differentially expressed hypoxic marker, CA9. As expected, a statistically significant negative association between CA9 expression, and tumor volume was observed for vehicle- and sorafenib-treated xenografts, while VEGF-treated xenografts displayed no relationship (Supplementary Figure S1). Taken together, these results suggest that modulating angiogenesis successfully modulates tumor growth and the extent of hypoxia within CAM cell line-derived xenografts. 

### 3.3. In Vivo Modulation of Hypoxia Affects Spontaneous Metastasis in the CAM Model

Intratumoral hypoxia is known to be associated with cancer progression and metastasis in various models as well as cancer patients [50,51]. To determine whether such association can also be found in the CAM xenograft model, HT-1080-derived xenografts were treated with angiogenesis-modifying agents, and primate Alu repeats were measured in the chick embryo livers of CAM bearing tumors. Interestingly, an increase in tumor hypoxia due to sorafenib treatment caused a significant increase in liver metastasis, whereas VEGF-induced decrease in hypoxia had the opposite effect (Figure 3D). This finding is in keeping with the fact that hypoxia can reduce cell–cell attachment, allowing cancer cells to invade, thereby increasing the risk of both local invasion and metastatic spread [6,7,8]. Overall, these results indicate that modulation of hypoxia levels within CAM xenografts can affect spontaneous metastasis in the chick embryo. 

### 3.4. Modulation of Hypoxia in ccRCC Patient-Derived CAM Xenografts

**Figure 4:**
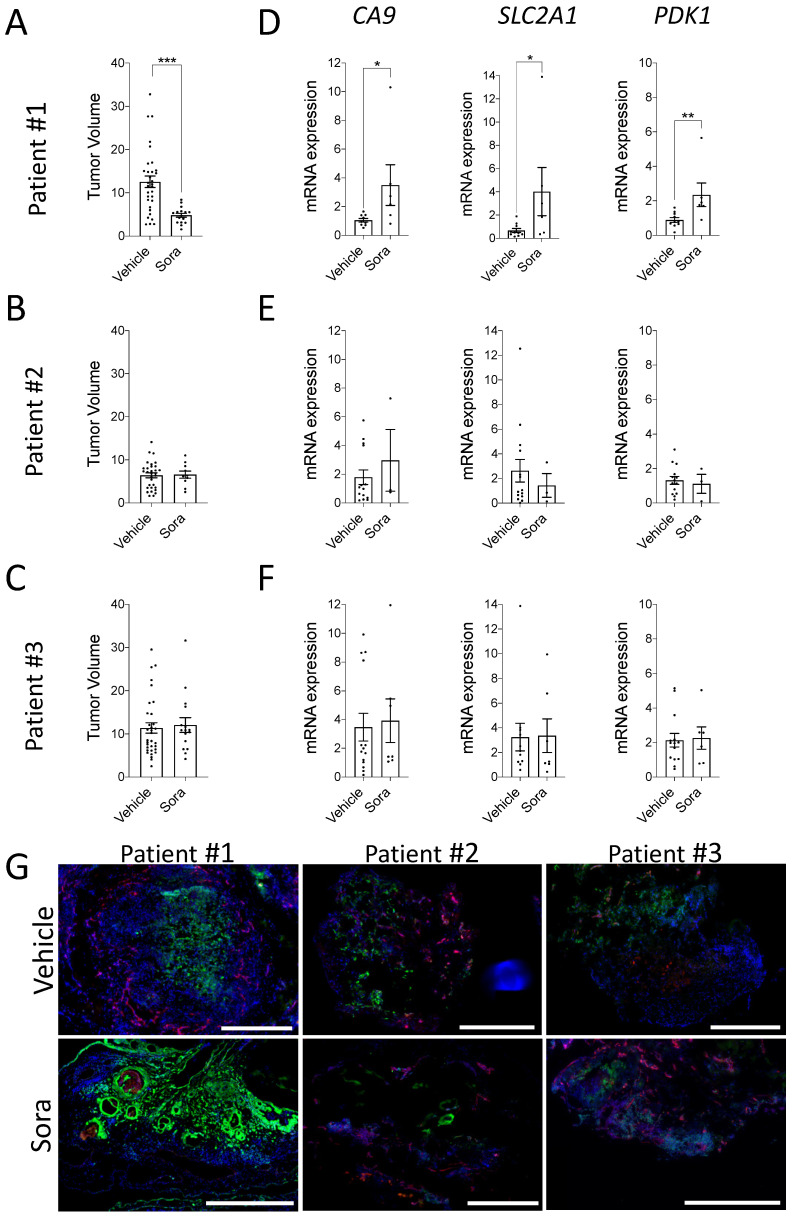
(**A**–**G**) Tumor fragments from human clear cell Renal Cell Carcinoma (ccRCC) patients were implanted onto the CAM and Sorafenib (Sora) or DMSO (vehicle) was added topically to the tumors every day. (**A**–**C**) Tumor volumes were calculated after 7 days of growth as the fold increase in tumor size from T0 to T1. *n* = 10–33 tumor fragments per group. (**D**–**F**) mRNA expression of *CA9* (CAIX), *SLC2A1* (GLUT1), and *PDK1* (PDK1) in Sorafenib (Sora) vs. DMSO (Vehicle) treated tumor xenografts is shown for 3 different patients. (**G**) Representative immunofluorescence images showing regions of hypoxia (pimonidazole; green) and vascularization (LCA; red) in DMSO (Vehicle) and Sorafenib (Sora) treated tumor xenografts from patient #1, #2, and #3 are shown. Nuclei were stained with DAPI (blue). Scale bar = 500 µm. Bars represent the mean ± SEM. The asterisks (*) correspond to *p* < 0.05 (*), *p* < 0.01 (**), and *p* < 0.001 (***), non-parametric *t* test.

To determine whether the above observations can be reproduced using patient-derived xenografts, tumor fragments from three different ccRCC patients were implanted onto the CAM, and xenografts were treated with vehicle or sorafenib for 7 days. Analysis of tumor volumes indicated a significant decrease in the mean volume of patient #1 xenografts, while xenografts derived from patients #2 and #3 failed to respond to the drug treatment (Figure 4A–C). Such a finding is consistent with the low response rates of ccRCC patients (9–15%) to sorafenib in the clinic [52,53]. We could not retrospectively analyze the clinical response to sorafenib as the three patients tested in the CAM assay did not receive sorafenib treatment. Of note, higher tumor size and gene expression variability were observed when using tumor fragment-derived xenografts compared to xenografts derived from cell lines (Figure 2A), an observation likely due to intra-tumoral heterogeneity of ccRCC tumors [54,55]. Next, the expression of common hypoxia-inducible genes (*CA9, SLC2A1*, and *PDK1)* was evaluated in vehicle- and sorafenib-treated ccRCC xenografts. Results indicated that the expression of hypoxia-responsive genes was significantly increased in sorafenib-responding patient #1 xenografts, while no significant modulation was observed for patients #2 and #3 (Figure 4D–F). Consistent with these results, similar pimonidazole staining was observed within CAM xenografts from patients #2 and #3, while for the sorafenib-responder patient, the enhanced hypoxia gene signature was associated with an increased abundance of hypoxic zones within the CAM xenograft (Figure 4G). These results demonstrate that hypoxia is present in ccRCC patient-derived CAM xenografts and can be modulated in selected patients.

## 4. Discussion

In the present study, we demonstrated that the CAM model recapitulates the hypoxic microenvironment characteristic of most types of cancer by xenografting various cancer cell lines and tumor tissues from ccRCC patients on the CAM. Results indicated that both patient and cell line-derived CAM xenografts contained elevated levels of hypoxia that can be modulated by pro- and anti-angiogenic treatments. Furthermore, the changes in hypoxia translated into modulated levels of metastasis, as measured by Alu-based assays.

Current in vivo models are essential for understanding the dynamic process of cancer progression, the identification of therapeutic targets, and the evaluation of anti-tumor drug efficacy. To this end, they must replicate, as closely as possible, the original tumor microenvironment, including restricted access to oxygen that results in intratumoral hypoxia. Hypoxia is a crucial element of the tumor microenvironment that is associated with tumor aggressiveness and resistance to treatments [2,56]. Due to the high metabolic demand and inadequate vasculature of such tumors, hypoxia is also a dynamic state that fluctuates according to the rapid cancer cell proliferation rate and the generation of new blood vessels within the tumor [48,57]. Previous reports have shown the presence of hypoxia in HT-1080 human fibrosarcoma and Renca mouse renal adenocarcinoma CAM xenografts [37,58]. Here we demonstrate that hypoxia is present within multiple cancer cell line-derived xenografts, as well as patient-derived ccRCC xenografts, when grown on the CAM. Hypoxic zones were found in both central and peripheral regions of the tumors and were localized in regions with less or absent vasculature. This is consistent with previous reports demonstrating heterogeneous distribution of hypoxic areas within the tumor mass, which arises as a result of an imbalance between oxygen supply and consumption [59,60,61]. Importantly, the CAM xenograft model also maintained the capacity to reproduce intratumoral hypoxic plasticity, as we found that angiogenesis modulators successfully altered the levels of hypoxia within cell line- and patient-derived CAM xenografts, as measured by the hypoxia marker pimonidazole and the expression of hypoxia-inducible genes.

Several key tumor progression-related cellular processes and signaling pathways are known to be activated in hypoxic cells. These include changes in gene expression that can promote angiogenesis and EMT [62,63], disrupted endocytosis, which can affect receptor activation and localization [64], and altered pH, leading to enhanced cell motility, ECM degradation, and drug resistance [37,65,66]. Likewise, many complex signaling networks interact with each other to mediate the effects of hypoxia, including the principal hypoxia-inducible factor (HIF) actor, PI3K/AKT, NF-kappaB, and MAPK pathways [67,68]. Despite these advances, the mechanisms underlying the response to hypoxia in the tumor microenvironment are still far from being completely understood [69]. In a recent publication, we found that a combination of inhibitors targeting hypoxia-induced receptor crosstalk successfully suppressed metastasis in a CAM-based fibrosarcoma model [38]. The same model was used to validate the biological relevance and target a pH-dependent mechanism involved in hypoxia-induced doxorubicin resistance [37]. Therefore, our demonstration of the widespread presence of hypoxia in a variety of CAM tumor models, along with the biologically relevant readout of metastasis, should provide an efficient and versatile model for testing therapeutic drugs targeting mechanisms involved in hypoxia-driven tumor progression.

Despite its well-known role in promoting tumor aggressiveness and resistance to treatment, intratumoral hypoxia remains a major obstacle in the treatment of cancer. Several hypoxia-selective drugs that target and kill hypoxic tumor cells have been developed, including nitramidazoles and hypoxia-activated pro-drugs such, as PR-104 [70,71]. However, no hypoxia-targeting drugs are currently approved for clinical use in humans. Various preclinical mouse models have been found to support the development of hypoxia in xenografts, and they are considered as the gold standard for preclinical testing of hypoxia-targeting drugs [14,15,16,17,18,19,20,21]. However, current mouse models are limited by high costs, lengthy experimental duration, and significant ethical considerations [25,72]. Furthermore, the results do not translate well to humans in clinical trials, explaining in part the failure of hypoxic pro-drugs to make it to market [72]. The CAM model provides an attractive alternative to mice as it is cost- and time-efficient allowing the assessment of tumor growth and metastasis within 5 to 7 days following inoculation [73,74]. Our finding, that CAM xenografts also successfully recapitulates the tumor hypoxic microenvironment in a various cancer cell lines and in patient-derived xenografts, suggests that the CAM model may provide a relevant preclinical platform to test the efficacy of hypoxia-activated pro-drugs in a time and cost-effective manner. Furthermore, compared to the low engraftment rate in mice [75], the success rate of implantation for human tumor fragments on CAM is at or near 100% for many different types of human cancers [76], indicating that the CAM model may allow testing of heterogenous patient tissues, thus providing a more accurate evaluation of drugs likely to succeed in human clinical trials.

## 5. Conclusions

In conclusion, the CAM xenograft assay has the potential to become a valuable model system for studying hypoxia and its influence on tumor progression and metastasis. Hypoxia is a tumor-promoting force driving cancer cell invasion that needs to be overcome in order to treat solid tumors and specifically block invasion and metastasis. The CAM xenograft model can help achieve this goal by providing an alternative cost and time-efficient model to test therapeutic interventions that target hypoxia-dependent processes.

## Data Availability

No new data were created or analyzed in this study. Data sharing is not applicable to this article.

## Supplementary Materials

The following are available online at https://www.mdpi.com/2072-6694/13/5/1093/s1, Figure S1: (A–C) Analysis of the correlation between tumor volume and CAIX expression in HT1080 CAM xenografts treated with DMSO (Vehicle), Sorafenib (Sora) or VEGF. Slope was determined by linear regression analysis. Spearman correlation was used to calculate r and *p* values, Video S1: Animated 3D reconstruction of CAM vasculature.
