# Supplementary material for: The Chicken Chorioallantoic Membrane Tumor Assay as a Relevant In Vivo Model to Study the Impact of Hypoxia on Tumor Progression and Metastasis
Source: Cancers (Basel). 2021 Mar 4;13(5):1093. doi: 10.3390/cancers13051093 (PMC7961795; doi:10.3390/cancers13051093)
Supplement: Supplementary file 1 [file cancers-13-01093-s001.zip › cancers-1111211 - supplementary/cancers-1111211 - supplementary -.docx]

Supplementary Materials

The Chicken Chorioallantoic Membrane Tumor Assay as a Relevant In Vivo Model to Study the Impact of Hypoxia on Tumor Progression and Metastasis

Kelly Harper, Anna Yatsyna, Martine Charbonneau, Karine Brochu-Gaudreau, Alexis Perreault, Claudio Jeldres, Patrick P. McDonald and Claire M. Dubois


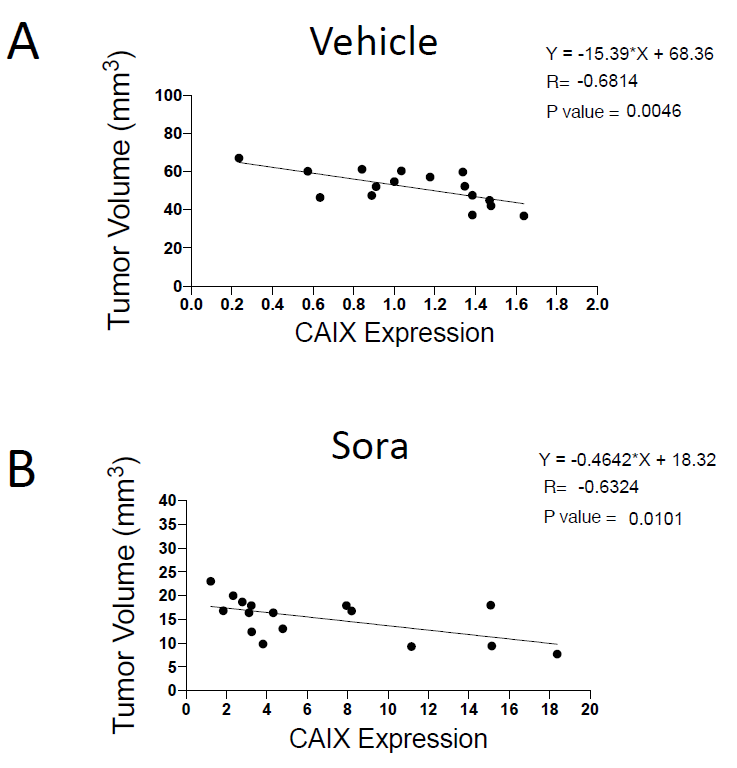

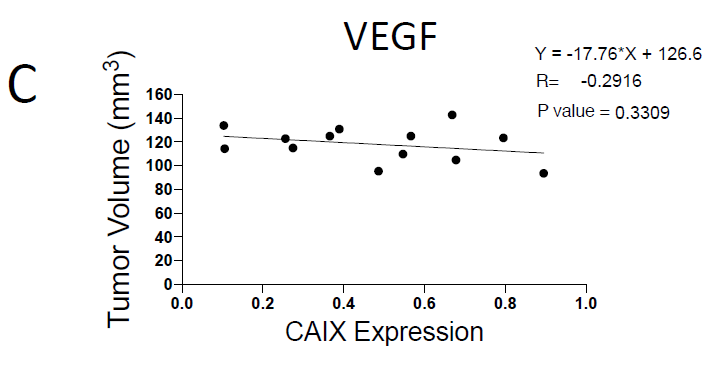


**Figure S1.** (A–C) Analysis of the correlation between tumor volume and CAIX expression in HT1080 CAM xenografts treated with DMSO (Vehicle), Sorafenib (Sora) or VEGF. Slope was determined by linear regression analysis. Spearman correlation was used to calculate *r* and *p* values.
